# Longitudinal analysis of hybrid immunity in a Vietnamese COVID-19 cohort demonstrates vaccination-associated broadening of cross-variant neutralization across diverse HLA backgrounds

**DOI:** 10.3389/fimmu.2026.1865246

**Published:** 2026-09-08

**Authors:** Thi Thanh Ngan Nguyen, Kim Mai Huynh, Hien Anh Thi Nguyen, Hung Thai Do, Kouichi Morita, Futoshi Hasebe, Thi Quynh Mai Le, Takashi Shiina, Lay-Myint Yoshida, Meng Ling Moi

**Affiliations:** 1 Graduate School of Biomedical Sciences, Nagasaki University, Nagasaki, Japan; 2 Department of Virology, Institute of Tropical Medicine, Nagasaki University, Nagasaki, Japan; 3 National Institute of Hygiene and Epidemiology, Hanoi, Vietnam; 4 Dejima Infectious Disease Research Alliance (DIDA), Nagasaki University, Nagasaki, Japan; 5 Pasteur Institute of Nha Trang, Nha Trang, Vietnam; 6 Department of Pediatric Infectious Diseases, Institute of Tropical Medicine, Nagasaki University, Nagasaki, Japan; 7 Vietnam Research Station, Center for Infectious Disease Researcher in Asia and Africa, Institute of Tropical Medicine, Nagasaki University, Nagasaki, Japan; 8 Department of Molecular Life Sciences, Tokai University School of Medicine, Kanagawa, Japan; 9 School of International Health, Graduate School of Medicine, The University of Tokyo, Tokyo, Japan; 10 The University of Tokyo Pandemic Preparedness (UTOPIA), Infection and Advanced Research Center, Tokyo, Japan

**Keywords:** antigenic cartography, cross-variant immunity, HLA polymorphism, hybrid immunity, immunogenetics, longitudinal cohort, Southeast Asia, vaccination

## Abstract

**Introduction:**

Despite extensive research on SARS-CoV-2 immunity, longitudinal studies integrating functional neutralization, antigenic relationships, and host genetic variability remain limited, particularly in Southeast Asian populations with distinct HLA backgrounds. Here, we performed a longitudinal analysis of a Vietnamese COVID-19 cohort before and after vaccination to characterize determinants of cross-variant antibody responses. Our findings indicate that while HLA polymorphisms contribute to inter-individual variability following natural infection, vaccination substantially enhanced cross-variant neutralization and reduced the apparent contribution of HLA-associated variability of neutralizing responses across genetically diverse individuals.

**Methods:**

We analyzed 189 Vietnamese COVID-19 convalescent individuals at approximately 7 months post-infection (unvaccinated) and 72 individuals at approximately 15 months (predominantly vaccinated). Anti-S1 and anti-RBD IgG were quantified by ELISA, and neutralizing antibody titers (PRNT_50_) were measured against Wuhan and major variants of concern. High-resolution HLA genotyping was performed using next-generation sequencing, and associations with antibody responses were evaluated using linear regression models with correction for multiple comparisons.

**Results:**

Vaccination markedly increased antibody levels and neutralizing titers across all variants, with fold-increases ranging from approximately 10-fold (Omicron) to 132-fold (Wuhan). Neutralization breadth expanded substantially after vaccination, although reduced activity against Omicron persisted. Strong correlations between binding and neutralizing antibodies were observed, particularly post-vaccination. While several HLA alleles were associated with variation in neutralizing antibody magnitude at earlier time points, multivariable analyses demonstrated that vaccination was the strongest factor associated with neutralization breadth at 15 months.

**Conclusion:**

Vaccination robustly enhances both the magnitude and breadth of humoral immunity in previously infected individuals, consistent with hybrid immunity. Although HLA polymorphisms contribute to inter-individual variability, their contribution appeared smaller than the effect associated with vaccination. These findings highlight how vaccination reshapes immune variability across genetically diverse populations and provide population-specific insight into long-term SARS-CoV-2 immunity.

## Introduction

Coronavirus disease 2019 (COVID-19), caused by severe acute respiratory syndrome coronavirus 2 (SARS-CoV-2), has resulted in profound global morbidity, mortality, and socioeconomic disruption since its emergence in late 2019 (1). The pandemic has been characterized by continuous viral evolution, leading to the emergence of multiple variants of concern (VOCs), including Alpha, Beta, Gamma, Delta, and the Omicron lineage. These variants differ in transmissibility, pathogenicity, and immune escape capacity, driven largely by mutations in the spike (S) protein that alter receptor binding and antibody recognition (2). The Delta variant was associated with increased viral load and disease severity (3, 4) whereas Omicron sublineages demonstrated enhanced transmissibility and substantial immune evasion, reflecting ongoing viral adaptation to rising population immunity (5, 6). As a result, the immunological landscape of SARS-CoV-2 infection has shifted from naïve exposure to complex patterns of immunity shaped by prior infection, vaccination, or both. In this context, hybrid immunity—defined as immunity acquired through a combination of natural infection and vaccination—has emerged as a dominant and highly protective immune state. Increasing evidence suggests that hybrid immunity confers broader and more durable protection than either infection-induced or vaccine-induced immunity alone, particularly against antigenically divergent variants (5, 7). This enhanced protection is thought to arise from repeated antigen exposure, leading to affinity maturation, expansion of memory B cells, and diversification of antibody repertoires. However, despite these general trends, substantial heterogeneity in immune responses persists across individuals, indicating that host-specific factors continue to shape the magnitude and breadth of immunity. In addition, the sequence of infection and vaccination exposures may contribute to immune imprinting effects that influence the development of memory B cell responses and the breadth of cross-reactive immunity.

Despite widespread vaccine deployment and the availability of antiviral therapies, recurrent waves of infection continue to occur globally, driven by waning antibody responses and viral immune escape (8, 9). Convalescent individuals—those who have recovered from SARS-CoV-2 infection—provide a critical framework for examining the durability and functional quality of immune responses over time. Antibodies targeting the spike (S) protein, particularly the S1 subunit and receptor-binding domain (RBD), are key correlates of protection and play a central role in viral neutralization (10). Longitudinal studies have demonstrated that while binding and neutralizing antibody levels decline over time, immunological memory can persist for months following infection (11–14). Nevertheless, the extent to which these responses confer cross-protection against newly emerging variants varies widely, highlighting the importance of understanding both viral and host determinants of immune heterogeneity. Among host factors, the human leukocyte antigen (HLA) system represents one of the most polymorphic and functionally important genetic determinants of antiviral immunity. HLA molecules are responsible for presenting pathogen-derived peptides to CD4^+^ helper and CD8^+^ cytotoxic T cells, thereby orchestrating adaptive immune responses and supporting antibody production (15–17). Variability in HLA class I (A, B, C) and class II (DR, DQ, DP) molecules influences peptide binding specificity and epitope presentation, which in turn can affect T-cell activation thresholds, clonal expansion, and downstream B-cell responses. In multiple viral infections, including HIV and hepatitis C virus, specific HLA alleles have been associated with differences in viral control and disease progression (18). In the context of COVID-19, emerging studies have linked specific HLA alleles to susceptibility, disease severity, and variability in immune responses following SARS-CoV-2 infection and vaccination. Several alleles, including HLA-A*11:01, HLA-C*04:01, and specific HLA-DRB1 variants, have been associated with differential clinical outcomes and antiviral immune responses (19–21). For example, HLA-C*04:01 *has been linked to increased disease severity, whereas other alleles such as* HLA-B*15:01 have been associated with asymptomatic infection and more effective viral clearance. Recent studies in Asian populations, including Vietnamese cohorts, have further suggested that population-specific HLA backgrounds may influence susceptibility to COVID-19 and the magnitude of adaptive immune responses. These observations highlight the importance of investigating HLA-associated immune heterogeneity across different ethnic populations, particularly in Southeast Asia where HLA distributions differ substantially from those reported in European populations (17, 19–21). Mechanistically, these associations may reflect differences in antigen presentation efficiency and the breadth of T-cell epitope recognition, ultimately influencing the quality and durability of humoral immunity (15, 22, 23). However, the extent to which HLA polymorphisms influence long-term antibody responses—particularly in the setting of vaccination—remains incompletely understood. While host genetic variation may shape early immune priming and the initial magnitude of antibody responses, repeated antigen exposure through vaccination has the potential to amplify and homogenize immune responses across individuals. This raises an important question as to what degree do genetic factors continue to contribute to immune variability once hybrid immunity is established. Addressing this question is critical for understanding inter-individual differences in vaccine responsiveness and for informing the design of broadly protective immunization strategies.

Importantly, HLA allele frequencies vary significantly across populations, reflecting evolutionary pressures and historical pathogen exposure (18, 24). Southeast Asian populations exhibit distinct HLA haplotypes—such as HLA-A*11:01, HLA-B*15:02, and HLA-C*01:02—that differ from those commonly observed in European cohorts (25). These population-specific genetic backgrounds may influence immune responses in ways that are not captured in studies conducted in other populations. Despite this, relatively few studies have examined the interplay between HLA polymorphisms and SARS-CoV-2 immune responses in Southeast Asian cohorts, particularly in a longitudinal framework that incorporates both pre- and post-vaccination time points. Vietnam provides a unique epidemiological setting to investigate these relationships. Early in the pandemic, the country implemented stringent public health measures, including aggressive contact tracing, quarantine, and travel restrictions, which effectively limited widespread transmission (26, 27). A localized outbreak in Da Nang in mid-2020 created a well-defined cohort of individuals infected prior to vaccine availability, offering a rare opportunity to study naturally acquired immunity in a largely SARS-CoV-2-naïve population. Subsequent waves of infection, driven by Alpha and Delta variants, led to the rapid rollout of vaccination programs, achieving high coverage by mid-2022. This sequence of events enables longitudinal assessment of immune responses before and after vaccination within the same population, providing a valuable framework for studying hybrid immunity.

Despite extensive global research on SARS-CoV-2 immunity, longitudinal studies integrating functional neutralization, antigenic mapping, and host genetic variation remain limited, particularly in Southeast Asian populations with distinct HLA backgrounds. Moreover, the extent to which vaccination modifies the contribution of host genetic factors on antibody responses in real-world settings remains incompletely understood. In this study, we conducted a longitudinal immunological and genetic analysis of 189 Vietnamese COVID-19 convalescent individuals approximately 7–12 months after infection, with follow-up evaluation of 72 samples at 15 months post-infection, including 68 paired longitudinal participants, most of whom had received vaccination. We quantified anti-SARS-CoV-2 S1 and RBD IgG levels, measured neutralizing antibody titers (PRNT_50_) against the ancestral Wuhan strain and major VOCs, and performed high-resolution HLA genotyping using next-generation sequencing. By integrating serological, neutralization, and genetic data across time points, we aimed to (i) characterize the durability and breadth of antibody responses following infection, (ii) assess the impact of vaccination on cross-variant neutralization in a real-world cohort, and (iii) define the relative contribution of HLA polymorphisms to inter-individual variability in antibody responses in the context of emerging hybrid immunity.

## Methods

### Study design and population

**Figure 1:**
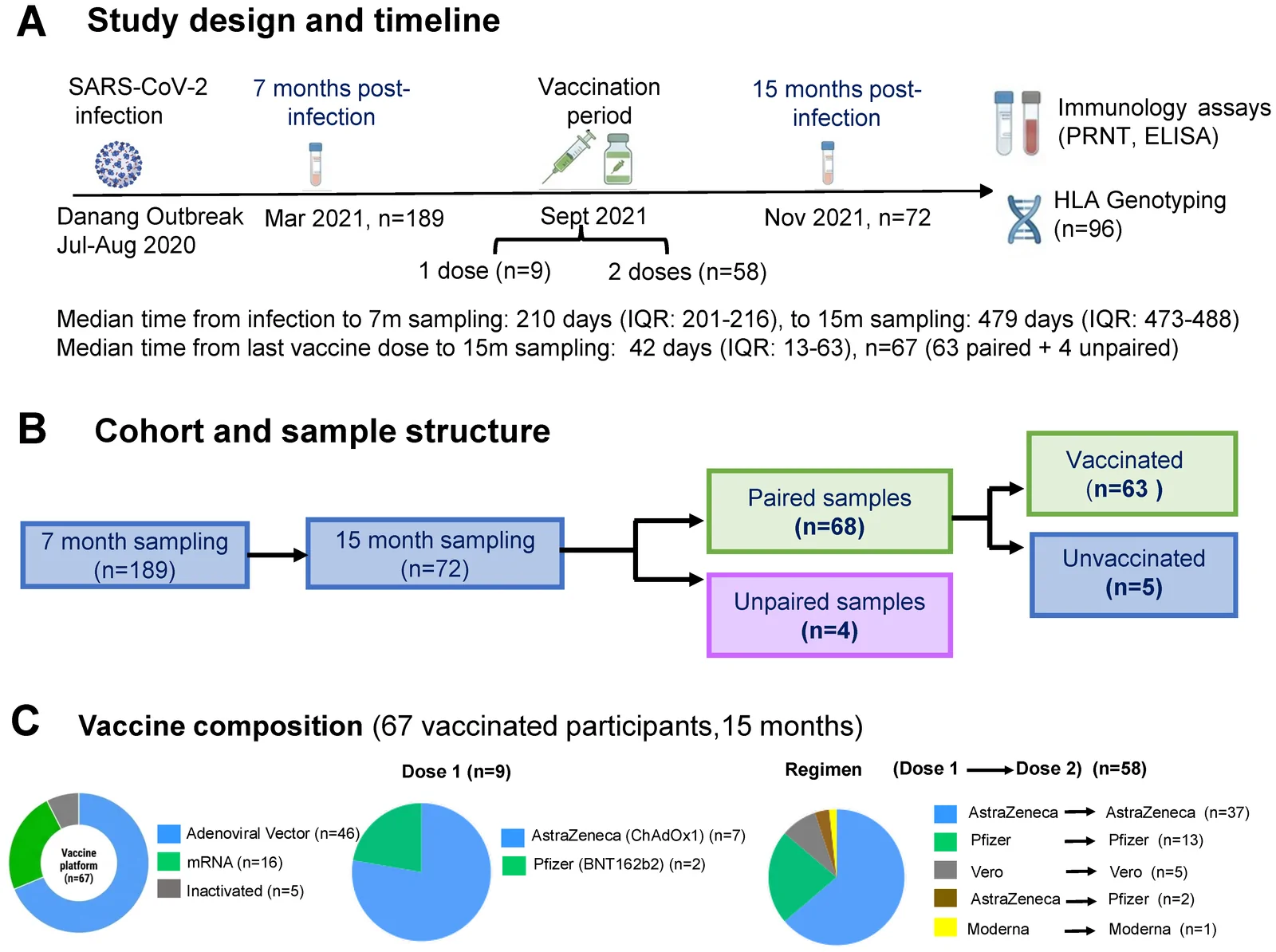
Study design, cohort structure, and vaccination profile of convalescent COVID-19 participants. **(A)** Schematic overview of the study timeline showing SARS-CoV-2 infection, longitudinal serum collection, vaccination period, and downstream immunological assays. Samples were collected at approximately 7 months and 15 months post-infection. Neutralizing antibody responses were evaluated by plaque reduction neutralization test (PRNT), and binding antibody responses were measured by ELISA. **(B)** Flow diagram of participant follow-up and longitudinal sample availability across the two collection time points. A total of 189 samples were collected at the first time point and 72 samples at the second time point, including 68 paired longitudinal samples and 4 unpaired samples available only at the 15-month follow-up. **(C)** Distribution of vaccine platforms, first-dose vaccines, and vaccination regimens among vaccinated convalescent participants sampled at the 15-month follow-up (n = 67).

This longitudinal study evaluated humoral immune responses and HLA-associated genetic factors in Vietnamese individuals who recovered from laboratory-confirmed SARS-CoV-2 infection during the Da Nang outbreak between July and August 2020. Because participants were recruited from a single outbreak wave prior to vaccine availability, the study was designed to evaluate long-term immune responses following a common primary SARS-CoV-2 infection event rather than repeated exposures. All participants were vaccine-naïve at the first collection, vaccine-platform stratification at 7 months represents retrospective grouping according to the vaccine subsequently received and should not be interpreted as a vaccine effect at baseline. The study design and cohort structure are summarized in **Figure 1**. Two follow-up collections were performed to assess the durability of infection-induced immunity and the impact of subsequent COVID-19 vaccination on antibody responses. The first collection comprised 189 serum samples obtained approximately 7 months after infection (median 210 days; interquartile range [IQR], 201–216), at which time all participants remained unvaccinated. A second collection was performed approximately 15 months after infection and included 72 serum samples (median 479 days; IQR, 473–488). Among these, paired longitudinal samples from both time points were available for 68 participants, whereas 4 samples were available only at the second collection. Because the paired subset was limited in size, additional multivariable sensitivity analyses restricted to paired participants were not performed. However, fold-rise analyses were restricted to paired vaccinated participants and showed consistent increases in neutralizing antibody titers across variants. Of the 68 participants included in the longitudinal follow-up cohort, 63 had received at least one dose of a COVID-19 vaccine before the second sampling, while 5 remained unvaccinated (**Figures 1A, B**). Vaccinated participants received one or more doses of mRNA vaccines (Pfizer-BioNTech BNT162b2 or Moderna mRNA-1273), viral-vector vaccines (ChAdOx1 nCoV-19, AstraZeneca), or inactivated whole-virus vaccines (Sinopharm BBIBP-CorV). The distribution of vaccine platforms is shown in **Figure 1C**. At each collection, serum samples were analyzed for anti-SARS-CoV-2 binding antibodies and neutralizing antibody responses against Wuhan and major variants of concern using ELISA and PRNT_50_ assays. High-resolution HLA genotyping was successfully performed for 96 participants for whom suitable whole-blood samples were available for genetic analysis (**Figure 1A**). Participants were classified as asymptomatic, mild, or severe/critical according to Vietnamese Ministry of Health guidelines based on clinical manifestations, oxygen saturation, respiratory status, and radiological findings. Demographic, clinical, and vaccination information was obtained through structured interviews and verified using available hospital records (**Table 1**).

**Table 1:** Anti-SARS-CoV-2 IgG levels based on participant characteristics.

Variable	7m (n)	αS1 IgG (log10​ ng/mL)	αRBD IgG (log10​ ng/mL)	15m (n)	αS1 IgG (log10​ ng/mL)	αRBD IgG (log10​ ng/mL)
Total	189	2.79 (2.61–3.03)	2.90 (2.79–3.05)	68	4.15 (3.72–4.61)	3.88 (3.44–4.27)
Age group						
≤ 15	8	2.91 (2.64–3.08)	2.91 (2.89–2.98)	2	4.36 (4.09–4.40)	4.03 (3.78–4.03)
16–30	39	2.66 (2.44–2.79)	2.84 (2.77–3.00)	8	4.10 (3.59–4.24)	3.72 (3.37–3.93)
31–45	44	2.82 (2.73–2.95)	2.88 (2.79–2.96)	18	4.10 (3.71–4.34)	3.86 (3.44–4.03)
46–60	54	2.79 (2.53–3.06)	2.92 (2.81–3.07)	23	3.99 (3.68–4.67)	3.81 (3.42–4.31)
> 60	44	2.89 (2.71–3.26)	2.93 (2.82–3.18)	17	4.58 (4.35–4.63)	4.15 (4.09–4.49)
Sex						
Male	75	2.70 (2.47–2.88)	2.85 (2.78–3.02)	30	4.06 (3.63–4.59)	3.86 (3.37–4.15)
Female	114	2.82 (2.67–3.05)	2.92 (2.82–3.07)	38	4.39 (3.91–4.62)	4.08 (3.69–4.32)
Clinical status						
Asymptomatic	126	2.77 (2.54–3.05)	2.90 (2.80–3.05)	49	4.13 (3.65–4.59)	3.87 (3.40–4.16)
Mild/Severe	63	2.82 (2.69–2.99)	2.90 (2.79–3.05)	19	4.41 (3.96–4.74)	4.18 (3.71–4.35)

Data is presented as median (interquartile range, IQR). IQR represents the 25th–75th percentile. Antibody concentrations are expressed in ng/mL.7m indicate 7 months, 15m indicate 15 months, α indicate anti.

Antigenic relationships among SARS-CoV-2 variants were inferred using antigenic cartography based on neutralization titers. PRNT_50_ values were arranged as a titer table with viruses defined as antigens and sera as columns and subsequently processed for antigenic map construction using the Racmacs package (Version 1.2.9).

### Sample collection and processing

Venous blood (5–10 mL) was collected from each participant and processed within two hours of sampling. Serum was separated by centrifugation at 1,000 × g for 10 minutes and stored at −80 °C until further analysis. Prior to neutralization testing, serum samples used for plaque reduction neutralization tests (PRNT) were heat-inactivated at 56 °C for 30 minutes to eliminate residual infectivity and complement activity. Whole-blood aliquots collected in EDTA tubes were stored at 4 °C for short-term preservation and processed for genomic DNA and RNA extraction within 24 hours for downstream HLA typing. All sample processing and neutralization assays were performed under biosafety level 3 (BSL-3) containment at the Institute of Tropical Medicine, Nagasaki University.

### Serologic quantification of anti-SARS-CoV-2 antibodies

Antibody quantification was performed using enzyme-linked immunosorbent assays (ELISA) targeting the SARS-CoV-2 Spike S1 and receptor-binding domain (RBD). Concentrations of anti-S1 and anti-RBD IgG were measured with the LEGEND MAX™ SARS-CoV-2 S1 Human IgG and RBD Human IgG ELISA kits (BioLegend Inc., USA) following the manufacturer’s instructions. Optical density was read at 450 nm using an Epoch Microplate Spectrophotometer (BioTek, USA), and antibody concentrations (ng/mL) were calculated from a standard curve generated using known IgG concentrations. Each assay plate included manufacturer-provided positive and negative controls according to the kit protocol to ensure assay consistency across batches.

### Plaque reduction neutralization test (PRNT_50_)

Neutralizing antibody (NAb) titers were determined using a 50% plaque-reduction neutralization test (PRNT_50_). Vero E6 cells (JCRB 9013) were seeded at a density of 2 × 10^4^ cells/well in 24-well plates and incubated overnight at 37 °C in 5% CO_2_. Heat-inactivated sera were serially twofold diluted from 1:10 until endpoint is reached, in Eagle’s Minimum Essential Medium (EMEM) supplemented with 2% fetal bovine serum (FBS) and mixed with equal volumes containing 62.5 plaque-forming units (PFU) of SARS-CoV-2. After incubation for one hour at 37 °C, virus–serum mixtures were added to cell monolayers and allowed to adsorb for one hour. The inoculum was removed and replaced with an overlay medium consisting of 1% methylcellulose in EMEM + 2% FBS, followed by incubation for 4–6 days. Plaques were visualized by crystal violet staining and manually counted. Neutralization titers were defined as the reciprocal serum dilution causing at least 50% reduction in plaque number compared with the virus-only control. A titer ≥ 1:160 was considered indicative of strong neutralizing capacity based on U.S. FDA criteria for convalescent plasma qualification (29). The SARS-CoV-2 isolates used in this study included Wuhan (hCoV-19/Japan/TY-WK-521/2020), Alpha (hCoV-19/Japan/QK002/2020), Beta (hCoV-19/Japan/TY8-612/2020), Gamma (hCoV-19/Japan/TY7-501/2021), Delta (hCoV-19/Japan/TY11-330-P1/2021), and Omicron BA.1 (hCoV-19/Japan/TY-38-873/2022). All assays were performed in duplicates, and mean values were used for analysis.

### HLA genotyping

High-resolution HLA genotyping was performed to characterize allelic diversity and its association with humoral immune responses. Total RNA was extracted from peripheral blood mononuclear cells (PBMCs) using the QIAamp RNA Blood Mini Kit (Qiagen, Germany) and reverse-transcribed into full-length complementary DNA (cDNA) to capture expressed HLA transcripts for high-resolution typing. Locus-specific primers were used to amplify the highly polymorphic regions of HLA-A, -B, -C, -DRB, -DQB, and -DPB genes with the Ion Express HLA Typing Kit (Thermo Fisher Scientific). Library preparation involved adapter and barcode ligation, magnetic-bead purification, and quantification with a Qubit fluorometer. Sequencing templates were generated using the Ion Chef System and paired-end sequencing was conducted on an Ion S5 platform with Ion 520/530 chips. Raw FASTQ reads were quality-filtered using fastp, and allele calls were assigned using the arcasHLA pipeline against the IMGT/HLA reference database (28). Only alleles supported by > 50× read depth and > 99% call confidence were included. All allele calls were confirmed by replicate sequencing to ensure consistency and accuracy. P-group designations were retained where allele calls corresponded to protein-level ambiguity groups according to IMGT/HLA nomenclature.

### Statistical analysis

Statistical analyses were conducted using GraphPad Prism version 10 (GraphPad Software, USA) and R version 4.4. (R Foundation for Statistical Computing, Vienna, Austria). Continuous variables were summarized as medians with interquartile ranges (IQR). Antibody concentrations were log10-transformed prior to analysis. Group comparisons were performed using the Kruskal–Wallis test with Dunn’s *post hoc* correction or the Wilcoxon signed-rank test for paired data. Correlations between IgG concentrations and neutralizing antibody titers (PRNT_50_) were evaluated using Spearman’s rank correlation coefficient. Statistical significance was defined as p < 0.05. Antigenic relationships among SARS-CoV-2 variants were analyzed using neutralization PRNT_50_ data obtained at 7 months and 15 months post-infection/vaccination. A total of 189 samples at 7 months and 72 samples at 15 months were included in the antigenic mapping analysis.

**Figure 2:**
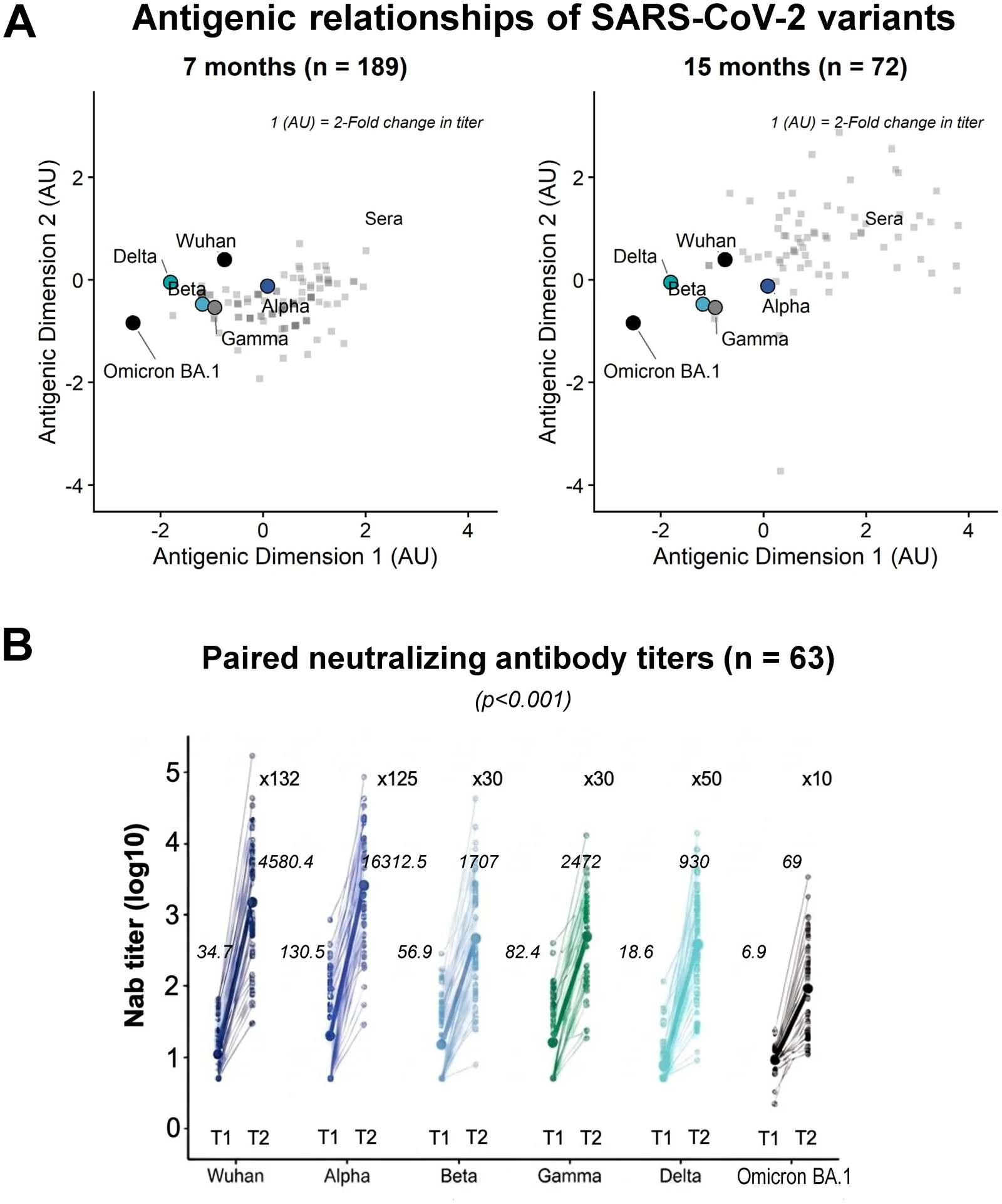
Antigenic relationships of SARS-CoV-2 variants and longitudinal antigenic landscape. **(A)** Antigenic maps of SARS-CoV-2 variants generated from PRNT50 neutralization titers at 7 months post-infection (n = 189) and 15 months post-infection/vaccination (n = 72). Antigenic maps were constructed using a shared coordinate framework based on the combined 7-month and 15-month PRNT50 titer matrices, allowing sera from each sampling time point to be displayed relative to the same antigenic landscape. Viruses are shown as colored circles and sera as grey squares. One antigenic unit (AU) corresponds to an approximately twofold change in neutralization titer. Shorter distances between sera and viruses indicate higher fitted neutralization titers, whereas greater distances indicate lower fitted neutralization titers. **(B)** Longitudinal changes in neutralizing antibody titers among vaccinated participants with paired samples (n = 63). PRNT50 titers are shown on a log_10_ scale for the first sampling time point (T1; 7 months post-infection) and the second sampling time point (T2; 15 months post-infection/vaccination). Semi-transparent lines indicate individual paired trajectories, and bold lines indicate geometric mean titers (GMTs). Numbers above each variant indicate the fold-rise in GMT between T1 and T2. The antigenic maps include all available samples at each time point, whereas the paired titer analysis is restricted to vaccinated participants with paired longitudinal samples.

Antigenic relationships among SARS-CoV-2 variants were inferred using antigenic cartography based on PRNT_50_ neutralization titers. PRNT_50_ values were organized into titer tables with viruses defined as antigens and individual sera as columns, and antigenic maps were constructed using the Racmacs package (version 1.2.9). For the revised **Figure 2A**, the 7-month and 15-month PRNT_50_ matrices were combined and optimized within a shared coordinate framework, allowing sera from each sampling time point to be displayed relative to the same antigenic landscape. Map optimization was performed in two dimensions, and goodness-of-fit was evaluated using the Racmacs stress function, which quantifies the discrepancy between observed and fitted titers. The combined shared-coordinate map, generated from 6 antigens and 261 sera, had a raw Racmacs stress of 1762.17, corresponding to a normalized root-mean-square error of approximately 1.06 antigenic units per fitted serum–virus titer relationship. This indicates that the fitted map reproduced the observed PRNT_50_ matrix with an average deviation of approximately one twofold dilution step, which is within the expected level of variation for neutralization assay-based antigenic mapping. As sensitivity checks, independently optimized 7-month and 15-month maps had raw stress values of 562.99 and 615.88, corresponding to normalized root-mean-square errors of approximately 0.70 and 1.19 antigenic units per fitted titer, respectively. Antigenic cartography was therefore interpreted as a complementary visualization of neutralization patterns, while direct PRNT_50_ titers provided the primary quantitative evidence.

Neutralizing antibody titers against SARS-CoV-2 variants, including Wuhan, Alpha, Beta, Gamma, Delta, and Omicron BA.1, were analyzed at two sampling time points (T1 and T2). Neutralization titers ≤10 were assigned a value of 1 prior to logarithmic transformation to avoid computational errors on the log scale. Neutralizing antibody responses were summarized as geometric mean titers (GMTs) with 95% confidence intervals (CI). GMTs were calculated from log-transformed titers and back-transformed to the original scale. For graphical and antigenic cartography analyses, PRNT_50_ values were estimated by interpolation from neutralization curves and treated as continuous IC_50_ values; therefore, plotted values are not restricted to the tested dilution endpoints. Fold-rise responses were calculated using paired samples (n = 63) as the ratio of GMTs between the second and first sampling time points (Fold rise = GMT_T2/GMT_T1). Confidence intervals for GMTs and fold changes were calculated on the log scale assuming a t-distribution and subsequently back-transformed. Neutralization breadth was defined as the number of SARS-CoV-2 variants with PRNT_50_ values above predefined neutralization thresholds. For infection-induced responses at 7 months, breadth was defined using PRNT_50_ ≥20, whereas for post-vaccination responses at 15 months, breadth was defined using PRNT_50_ ≥160. These thresholds were selected to distinguish detectable neutralization from stronger neutralizing activity after vaccination.

Associations between HLA alleles and antibody responses were evaluated using linear regression models with log10-transformed antibody titers as the dependent variable and allele presence as the independent variable. Effect sizes are reported as β coefficients with standard errors (SE) and corresponding fold changes with 95% confidence intervals (CI). Multiple testing correction was performed using the Benjamini–Hochberg false discovery rate (FDR) method. Neutralization breadth at 7 and 15 months post-infection was analyzed using multivariable linear regression models adjusted for vaccination status, age, and sex. Associations between HLA alleles (frequency ≥10%) and categorical variables (age group, sex, and clinical status) were assessed using Fisher’s exact test, with statistical significance defined as p < 0.05.

## Results

A total of 189 convalescent participants were analyzed at approximately 7 months post-infection, and 68 of these individuals were re-evaluated longitudinally at 15 months post-infection to assess long-term immune dynamics (**Figure 1B**). Of the 68 follow-up participants, 63 had received at least one dose of a COVID-19 vaccine within two months prior to the second sampling, whereas 5 participants remained unvaccinated. Vaccinated participants received either mRNA (Pfizer-BioNTech or Moderna), viral-vector (AstraZeneca), or inactivated (Sinopharm BBIBP-CorV) vaccines. The first sampling time point represented the pre-vaccination phase of the pandemic in Vietnam, whereas the second sampling included individuals with hybrid immunity following infection and subsequent vaccination. The median age of the cohort was 42 years (range, 6–82 years), and 60% were female. Most infections were mild, with only a small proportion requiring hospitalization during the Da Nang outbreak. Demographic and clinical characteristics are summarized in **Table 1**.

### Binding antibody responses

**Figure 3:**
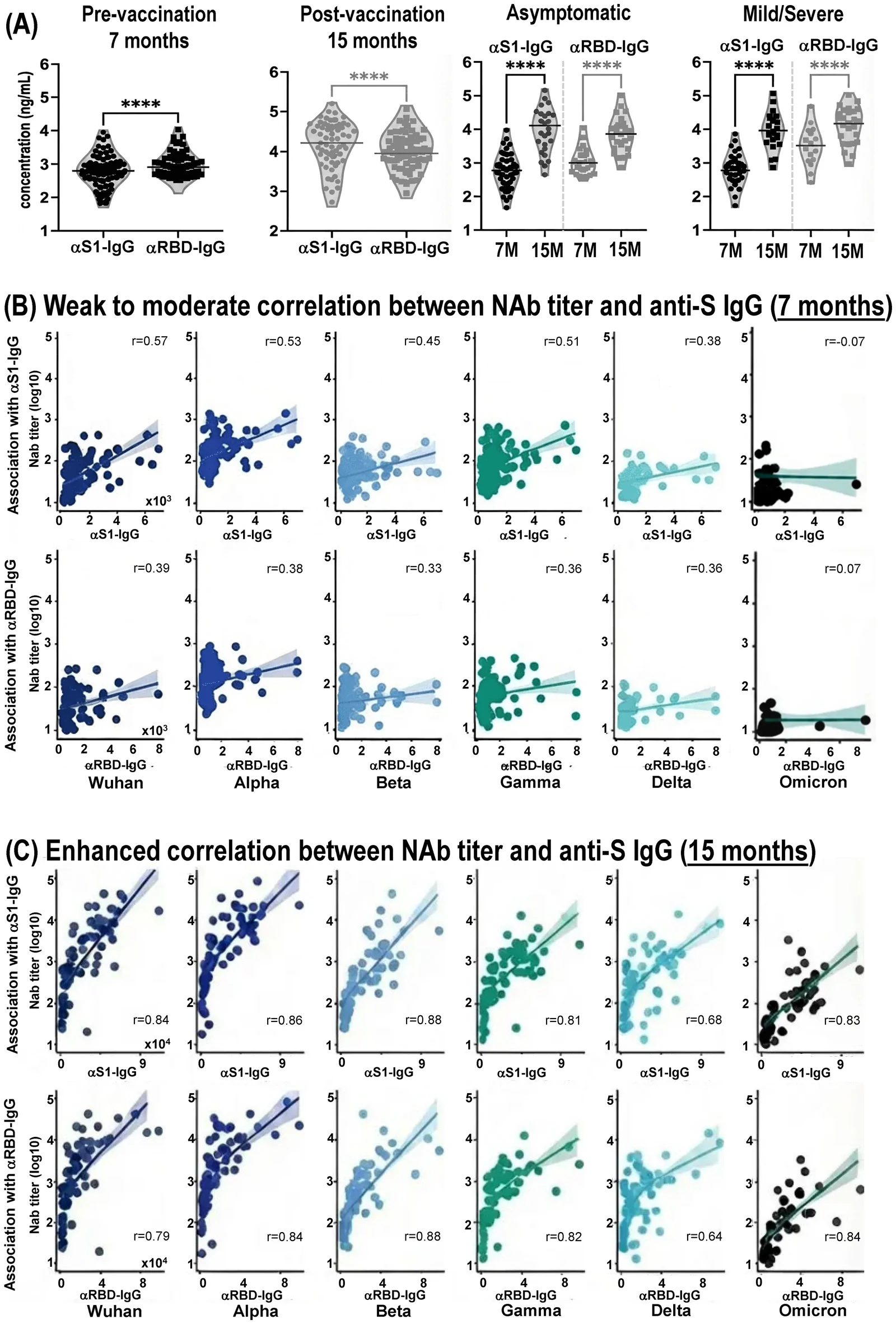
Correlation between binding and neutralizing antibody responses. **(A)** Anti-S1 IgG and anti-RBD IgG concentrations at 7 and 15 months stratified by clinical status. Data are presented as log10-transformed concentrations (ng/mL). **(B)** Correlation between anti-S1 and anti-RBD IgG levels and neutralizing antibody titers (PRNT_50_) at 7 months. Each point represents an individual participant; lines indicate linear regression with 95% confidence intervals. **(C)** Correlation between anti-S1 and anti-RBD IgG levels and neutralizing antibody titers (PRNT_50_) at 15 months. Stronger and more consistent correlations are observed following vaccination across all variants.

At seven months post-infection, the median log10 concentrations of anti-Spike S1 and anti-RBD IgG were 2.79 (IQR: 2.61–3.03) and 2.90 (IQR: 2.79–3.05), respectively. By 15 months, these values increased to 4.15 (IQR: 3.72–4.61) for anti-S1 IgG and 3.88 (IQR: 3.44–4.27) for anti-RBD IgG (**Table 1**). These findings suggest partial waning of infection-induced immunity followed by substantial enhancement after vaccination. Following vaccination, IgG titers increased markedly in nearly all participants. Median anti-S1 IgG increased approximately 23-fold, while anti-RBD IgG increased approximately 10-fold (Wilcoxon signed-rank test, p < 0.001 for both comparisons; **Figure 3**). Stratified analyses by age, sex, and clinical status revealed considerable inter-individual variability (**Table 1**). Female participants and individuals aged >60 years tended to exhibit higher median antibody levels compared with male or younger participants, although these differences were not statistically significant. Participants with symptomatic infection showed higher antibody levels than asymptomatic individuals, particularly at the 15-month time point. Differences across age groups and clinical categories were evaluated using the Kruskal–Wallis test. Exploratory stratified comparisons were performed, but no statistically significant differences were observed.

### Neutralizing antibody activity

Antigenic cartography revealed distinct changes in the antigenic landscape of SARS-CoV-2 variants between the two sampling time points (**Figure 2A**). At the first sampling time point (7 months post-infection), Wuhan, Beta, and Gamma variants clustered relatively close together, indicating substantial antigenic similarity and cross-reactive neutralizing responses. The Alpha variant showed moderate separation from the central cluster, whereas Delta and, particularly, Omicron BA.1 were positioned further away, suggesting greater antigenic divergence and reduced susceptibility to infection-induced neutralizing antibodies. At the second sampling time point (15 months post-infection), following vaccination in most participants, the antigenic landscape became more broadly distributed. Neutralizing responses against Beta, Gamma, and Delta variants showed reduced antigenic separation compared with the first time point, indicating expansion of cross-reactive immunity after hybrid immune exposure. However, Omicron BA.1 remained the most antigenically distant variant, consistent with persistent immune escape despite vaccination-induced boosting.

Longitudinal analysis of paired sera (n = 63) demonstrated marked increases in neutralizing antibody titers against all tested variants following vaccination (**Figure 2B**). The greatest fold increases were observed against Wuhan (×132.4) and Alpha (×125.7), whereas lower fold rises were detected against Beta (×30.2), Gamma (×30.1), Delta (×50.9), and especially Omicron BA.1 (×10.4). These findings indicate that vaccination substantially enhanced both the magnitude and breadth of neutralizing antibody responses, although antigenically distant variants such as BA.1 remained relatively resistant to neutralization. Collectively, these results suggest that hybrid immunity reshaped the SARS-CoV-2 antigenic landscape by broadening cross-neutralizing activity across variants while maintaining reduced effectiveness against highly divergent Omicron lineages.

Among vaccine platforms, mRNA vaccines elicited the highest antibody responses, followed by viral-vector and inactivated vaccines. Variant-specific neutralizing responses stratified by vaccine platform are shown in **Supplementary Figure 1**. No significant difference was observed between single- and double-dose recipients, suggesting that recent antigenic exposure rather than dose number was the primary determinant of antibody magnitude.

At the first collection (7 months post-infection, n = 189), neutralizing activity was highest against the ancestral Wuhan and Alpha strains, followed by Gamma and Beta variants, whereas reduced titers were observed against Delta and Omicron BA.1 (**Supplementary Figure 2A**). Overall, differences in neutralization titers were observed among variants, with Omicron BA.1 showing the lowest neutralizing responses compared with ancestral and earlier variants.

Following vaccination, a substantial increase in neutralizing antibody titers was observed against all tested variants at the second collection (overall 15-month cohort, n = 72; paired longitudinal subset, n = 68) (**Supplementary Figure 2B**). Despite this overall enhancement, neutralization titers against Omicron BA.1 remained lower than those against Wuhan, Alpha, and Delta variants, indicating a persistent reduction in neutralization capacity against BA.1 even after hybrid immune exposure. In the paired vaccinated subset (n = 63; **Supplementary Figure 2C**), statistically significant differences were observed only between the Wuhan and Alpha variants, whereas no other pairwise comparisons reached statistical significance. To further visualize the distribution of neutralizing antibody titers across variants, PRNT_50_ titers were categorized as <20, 20–80, 80–160, and ≥160 (**Supplementary Figure 3**). This distribution analysis showed a marked increase in the proportion of high-titer responses at 15 months compared with 7 months, while Omicron BA.1 retained a larger proportion of low-titer responses than the pre-Omicron variants.

Stratified analyses according to clinical presentation showed that symptomatic individuals tended to exhibit higher neutralizing antibody titers than asymptomatic participants at both sampling time points (**Supplementary Figures 2D, E**). At the first collection, variant-specific differences were observed within both asymptomatic and mild/severe groups, with consistently higher neutralization against Wuhan and Alpha variants. At the second collection, vaccination markedly enhanced neutralizing responses across all clinical groups; however, Omicron BA.1 consistently exhibited the lowest neutralization titers irrespective of disease severity. The proportion of sera achieving high neutralization (log_10_ PRNT_50_ ≥ 2.2, or PRNT_50_ ≥160) increased markedly from 20.6% before vaccination to 85.5% after vaccination.

### Correlation between binding and neutralizing responses

Strong positive correlations were observed between anti-S1 or anti-RBD IgG concentrations and neutralizing antibody titers across all variants (Spearman’s ρ = 0.76 for S1; ρ = 0.71 for RBD; p < 0.001) (**Figures 3B, C**). Prior to vaccination, correlations were moderate and varied by variant, with weaker associations observed for Omicron. Following vaccination, correlations became stronger and more consistent across all variants. Linear regression analysis showed that post-vaccination IgG levels accounted for approximately 60% of the variance in neutralizing titers (**Figure 3C**), indicating that binding antibody levels correlated with neutralizing activity, although they should not be considered substitutes for variant-specific neutralization assays, particularly for antigenically divergent variants such as Omicron.

### HLA allele distribution and associations with antibody responses

High-resolution HLA genotyping identified 192 distinct alleles across class I and class II loci. The distribution of common alleles (frequency ≥10%) is summarized in **Table 2**. The most frequent alleles included HLA-A*11:01, HLA-A*24:02, HLA-B*15:02, HLA-C*01:02, HLA-C*07:02, HLA-DRB1*12:02P, HLA-DQB1*03:01P, and HLA-DPB1*05:01, consistent with known Southeast Asian haplotypes. Associations between HLA alleles and antibody or neutralizing responses were evaluated using linear regression models with log10-transformed antibody titers (**Table 3**). Several alleles were associated with increased neutralizing responses, including HLA-C*01:02, HLA-DRB1*12:02, and HLA-DQB1*03:01P, whereas HLA-A*02:03 and HLA-DRB1*12:02P were associated with reduced responses. These associations were statistically significant at the level of raw p-values but were attenuated after multiple testing correction using the Benjamini–Hochberg false discovery rate (FDR) (**Table 3**), indicating modest effect sizes.

**Table 2:** Common HLA alleles (frequency ≥10%) in the study population.

Locus	Allele	Allele count (n)	Frequency (%)
HLA-A	A*11:01	48	25
	A*24:02	30	16
	A*33:03	23	12
	A*02:03	19	10
HLA-B	B*15:02	21	11
	B*46:01	19	10
HLA-C	C*01:02	33	17
	C*07:02	28	15
	C*08:01	19	10
HLA-DRB	DRB1*12:02P	34	18
	DRB1*12:02	23	12
HLA-DQB	DQB1*03:01P	65	34
	DQB1*03:03P	24	13
	DQB1*05:01P	20	10
HLA-DPB	DPB1*05:01	29	15
	DPB1*05:01P	29	15

Only alleles with frequency ≥10% are shown. P indicates pseudoalleles; alleles with identical counts reflect typing resolution. P-group designations were retained where allele calls corresponded to protein-level ambiguity groups according to IMGT/HLA nomenclature.

**Table 3:** Association between HLA alleles and antibody and neutralizing responses.

HLA locus	Allele	Beta ± SE	Fold change (95% CI)	FDR	N (frequency)
Anti-S1 IgG (7m)					
HLA-A	A*02:03	-0.29 ± 0.13	0.52 (0.29–0.91)	0.641	16 (0.17)
HLA-A	A*24:02	-0.35 ± 0.15	0.44 (0.23–0.86)	0.555	19 (0.20)
Anti-RBD IgG (7m)					
HLA-B	B*13:01	0.30 ± 0.11	1.98 (1.21–3.24)	0.555	13 (0.14)
HLA-DPB1	DPB1*05:01P	0.21 ± 0.07	1.62 (1.16–2.27)	0.555	21 (0.22)
HLA-DRB1	DRB1*09:01P	0.25 ± 0.10	1.77 (1.14–2.74)	0.555	11 (0.11)
Wuhan PRNT_50_					
HLA-A	A*02:03	-0.46 ± 0.17	0.35 (0.16–0.73)	0.555	16 (0.17)
HLA-C	C*01:02	0.36 ± 0.15	2.28 (1.16–4.47)	0.555	31 (0.32)
HLA-DQB1	DQB1*03:01P	0.47 ± 0.22	2.98 (1.11–8.02)	0.679	20 (0.21)
HLA-DRB1	DRB1*12:02	0.73 ± 0.24	5.33 (1.76–16.08)	0.530	13 (0.14)
Alpha PRNT_50_					
HLA-A	A*02:03	-0.71 ± 0.29	0.19 (0.05–0.73)	0.555	16 (0.17)
HLA-DQB1	DQB1*03:01P	0.79 ± 0.38	6.15 (1.09–34.66)	0.689	20 (0.21)
HLA-DRB1	DRB1*12:02P	-0.74 ± 0.36	0.18 (0.04–0.91)	0.679	14 (0.15)
HLA-DRB1	DRB1*12:02	0.95 ± 0.45	8.90 (1.17–67.53)	0.679	13 (0.14)
Beta PRNT_50_					
HLA-DQB1	DQB1*03:01P	0.79 ± 0.26	6.17 (1.91–19.91)	0.521	20 (0.21)
HLA-DRB1	DRB1*12:02	0.81 ± 0.32	6.47 (1.56–26.83)	0.555	13 (0.14)
Delta PRNT_50_					
HLA-A	A*33:03	0.32 ± 0.15	2.11 (1.08–4.15)	0.675	21 (0.22)
HLA-C	C*03:02	0.48 ± 0.20	2.99 (1.23–7.27)	0.555	12 (0.13)
HLA-DQB1	DQB1*03:01P	0.47 ± 0.16	2.94 (1.42–6.07)	0.546	20 (0.21)
HLA-DRB1	DRB1*12:02	0.56 ± 0.19	3.63 (1.55–8.49)	0.53	13 (0.14)
Gamma PRNT_50_					
HLA-A	A*02:03	-0.67 ± 0.21	0.21 (0.08–0.56)	0.481	16 (0.17)
HLA-DQB1	DQB1*03:01P	0.78 ± 0.28	6.08 (1.74–21.29)	0.555	20 (0.21)
HLA-DRB1	DRB1*12:02P	-0.62 ± 0.27	0.24 (0.07–0.80)	0.608	14 (0.15)
Omicron PRNT_50_					
HLA-B	B*46:01	0.16 ± 0.06	1.44 (1.08–1.92)	0.555	15 (0.16)
HLA-C	C*01:02	0.13 ± 0.06	1.35 (1.03–1.77)	0.675	31 (0.32)
HLA-DPB1	DPB1*02:01	0.19 ± 0.08	1.54 (1.09–2.19)	0.555	14 (0.15)
HLA-DRB1	DRB1*12:02	0.25 ± 0.09	1.78 (1.17–2.70)	0.555	13 (0.14)

Beta +/- SE, effect size +/- standard error; Fold Change (95% CI), fold change with 95% confidence interval.

FDR, Benjamini-Hochberg adjusted p-value; N (Freq), number of alleles and frequency. P indicates pseudoalleles; alleles with identical counts reflect typing resolution. Positive β indicates increased antibody or neutralizing response, while negative β indicates decreased response.

### Multivariable analysis of neutralization breadth

Neutralization breadth at 7 and 15 months post-infection was analyzed using multivariable linear regression models adjusted for vaccination status, age, and sex (**Supplementary Table 1**). At 7 months, neutralization breadth showed a modest association with age (β = 0.026 per year, p = 0.036), whereas vaccination status and sex were not significantly associated. In contrast, at 15 months, vaccination was the strongest factor associated with neutralization breadth (β = 4.07, p = 1.3 × 10^-^²^9^), while age and sex were not associated. Comparative analysis of pre- and post-vaccination data demonstrated that vaccination substantially enhanced humoral immunity in previously infected individuals. Antigenic mapping confirmed that hybrid immunity broadened cross-variant recognition while maintaining reduced neutralization against Omicron (**Figure 2B**). Collectively, these findings demonstrate that vaccination is one of the stronger factors associated with neutralization breadth in this cohort, while HLA polymorphisms contribute to inter-individual variability in antibody responses (**Table 3**; **Supplementary Table 1**).

## Discussion

This study provides a longitudinal evaluation of humoral immune responses and HLA-associated genetic factors to SARS-CoV-2 infection and subsequent vaccination in a Vietnamese cohort, offering insight into the dynamics of hybrid immunity in an underrepresented population. We demonstrate that vaccination substantially enhances both the magnitude and breadth of humoral immunity following prior SARS-CoV-2 infection, consistent with the concept of hybrid immunity across multiple variants of concern. These findings are consistent with previous studies demonstrating durable but gradually waning immune memory following natural infection (14, 29) as well as strong recall responses following vaccination in convalescent individuals (30, 31). Our findings provide new insight into the interaction between host genetic variability and vaccine-induced immunity by demonstrating that vaccination enhanced cross-variant neutralization and reduced the apparent contribution of HLA-associated variability of neutralizing responses across individuals with diverse HLA backgrounds. This effect is particularly notable in a Southeast Asian cohort, where HLA allele distributions differ substantially from those reported in predominantly European populations.

Neutralizing antibody responses in this cohort exhibited clear variant-dependent patterns that reflect ongoing viral evolution. Prior to vaccination, neutralization capacity was strongest against early circulating strains such as Wuhan and Alpha, with progressively reduced activity against later variants including Beta, Gamma, Delta, and especially Omicron (32). These observations are consistent with the accumulation of spike mutations that enhance viral fitness and immune evasion (2–4), as well as empirical studies demonstrating reduced neutralization sensitivity of emerging variants (12, 13, 33). Following vaccination, neutralizing titers increased substantially across all tested variants, confirming the ability of booster immunization to broaden cross-reactive antibody responses. However, neutralization of Omicron remained comparatively lower, even after vaccination, highlighting the antigenic divergence of this variant and its sublineages. This is consistent with multiple reports showing that Omicron variants exhibit considerable escape from neutralizing antibodies elicited by prior infection or vaccination (6, 34).

A central finding of this study is that vaccination was the strongest factor associated with neutralizing antibody breadth at later time points in this cohort. While modest associations with age were observed in the infection-only phase, these effects were largely diminished following vaccination, and no consistent association was identified with sex. These results reinforce the concept that repeated antigen exposure through vaccination is one of the stronger measured factors associated with antibody magnitude and breadth (30, 31) and are further supported by meta-analytic evidence demonstrating the effectiveness and durability of booster vaccination strategies across populations. The strong correlation observed between binding antibody levels and neutralizing activity, particularly after vaccination, is also consistent with prior studies identifying antibody titers as a key correlate of protection against SARS-CoV-2 infection (33).

Together, these findings suggest that vaccination appeared to reduce the relative influence of demographic variability and enhance functional antibody responses in previously infected individuals. Nevertheless, host genetic factors may still contribute to inter-individual variability in immune responses. Recent studies in Vietnamese populations have identified HLA alleles associated with susceptibility and severity of COVID-19 (35). In addition to these vaccination-driven effects, we identified associations between specific HLA alleles and variation in neutralizing antibody responses, particularly at earlier time points following infection. These findings are biologically plausible given the central role of HLA molecules in antigen presentation and the activation of adaptive immune responses, including T-cell-mediated help for B-cell antibody production (15, 22). Previous studies have reported associations between HLA polymorphisms and both susceptibility to SARS-CoV-2 infection and disease severity (19–21), as well as differences in antibody responses (17), supporting the relevance of host genetic variation in shaping immune outcomes. In the present study, however, the magnitude of HLA-associated effects was modest compared with the impact of vaccination, and these associations appeared to diminish following booster immunization. This pattern suggests that while HLA polymorphisms may influence the initial priming and early magnitude of immune responses, their relative contribution becomes less pronounced following repeated antigen exposure and immune expansion.

This attenuation of genetic effects in the context of vaccination is consistent with broader immunological principles and observations in other viral infections, where HLA-associated variability is often more evident during primary immune responses than during secondary or memory responses (18, 22). It is also possible that vaccine-induced responses, particularly those elicited by mRNA platforms, may engage a broader repertoire of epitopes and immune pathways, thereby reducing the dependence on specific HLA-restricted antigen presentation. Furthermore, the diversity of vaccine platforms represented in this cohort, including mRNA, viral vector, and inactivated vaccines, may contribute to heterogeneity in immune responses, although the overall effect of vaccination remains important. Antigenic cartography has been increasingly used to visualize antigenic relationships among SARS-CoV-2 variants and quantify antigenic distance (38). The antigenic cartography analysis further supports these findings by illustrating how vaccination reshapes the antibody landscape. Antigenic cartography provided a complementary visualization of the broadening of cross-reactive immunity following vaccination. Although cross-neutralization improved across most variants, Omicron BA.1 remained antigenically distinct, consistent with persistent immune escape. These observations are consistent with recent studies employing antigenic mapping approaches to characterize SARS-CoV-2 evolution and immune escape (37) and highlight the importance of continued surveillance and potential vaccine updating strategies. Additionally, the broadening of neutralization observed following vaccination may also reflect immune imprinting and recall of infection-induced memory B-cell populations, which have been shown to contribute to hybrid immunity (5, 31). These findings are consistent with the concept of hybrid immunity, whereby sequential exposure through infection and vaccination generates broader immune responses than either exposure alone (39).

An important strength of this study is the focus on a Vietnamese cohort, providing data from a population that is underrepresented in global immunological and genetic studies. HLA allele frequencies vary substantially across geographic regions and ethnic groups, and such variation can influence both immune responses and disease outcomes (24, 36). Our findings therefore contribute valuable population-specific insights into the interplay between host genetics and vaccine-induced immunity. Similar enhancement of breadth has been observed in Europe, North America and Israel following hybrid immunity, although our findings extend these observations to a Southeast Asian population with distinct HLA distributions (5, 30, 31). In particular, the identification of HLA-associated differences in antibody responses may inform future studies on vaccine responsiveness and immune profiling in diverse populations. However, these findings should be interpreted with caution, as the sample size for certain subgroup analyses was limited, and replication in larger cohorts will be necessary to confirm these associations.

Several limitations should be considered when interpreting these results. First, the post-vaccination sample size was relatively modest, which may limit the statistical power to detect weaker associations, particularly in genetic analyses. Second, this study focused on humoral immune responses and did not include direct assessment of cellular immunity, which plays a critical role in protection against severe disease and may also be influenced by HLA polymorphisms. Third, neutralization assays were performed against a limited panel of variants, including Omicron BA.1, and did not encompass more recently circulating subvariants with additional immune escape mutations. An additional limitation is the use of multiple vaccine platforms, including mRNA, viral-vector, and inactivated vaccines, which may introduce heterogeneity in immune responses. Because the second collection occurred approximately two months after vaccination but fifteen months after infection, comparisons between time points reflect both waning of infection-induced immunity and boosting by recent vaccination. In this context, due to the small number of participants in some vaccine-platform subgroups, particularly the inactivated vaccine group, binding antibody comparisons across vaccine platforms were not statistically robust and should be interpreted cautiously. After correction for multiple comparisons, most HLA associations did not remain statistically significant, indicating that these effects should be interpreted as exploratory and hypothesis-generating rather than definitive. Due to sample size limitations, multivariable adjustment within HLA association models was limited, and residual confounding by factors such as vaccine platform and timing cannot be excluded. However, this diversity reflects real-world vaccination practices and provides a broader perspective on hybrid immunity in the population. Although mRNA vaccines were associated with higher antibody responses, the overall effect of vaccination on neutralization breadth was consistent across platforms, indicating that the observed findings are robust across different immunization strategies. Because of the limited sample size and uneven distribution of vaccine platforms, number of doses, and time since most recent vaccination, these factors could not be fully incorporated into the primary multivariable models. Therefore, the observed association between vaccination status and neutralization breadth should be interpreted as reflecting vaccination-associated immune boosting in this cohort, rather than as evidence that vaccination status alone accounts for all variation in neutralization breadth. Finally, while associations between HLA alleles and antibody responses were identified, mechanistic studies were not performed to directly link specific alleles to functional immune outcomes. Despite these limitations, the longitudinal design, inclusion of paired samples, and comprehensive analysis of neutralizing responses across multiple variants strengthen the robustness of our findings. By integrating serological, neutralization, and immunogenetic data, this study provides a multidimensional view of SARS-CoV-2 immune responses in a real-world setting.

In conclusion, our results demonstrate that vaccination showed strongest association with durable and broadly reactive neutralizing antibody responses following SARS-CoV-2 infection, consistent with the concept of hybrid immunity. While HLA polymorphisms contribute to inter-individual variability in antibody magnitude, their influence is secondary to the effects of vaccination. These findings highlight the important role of vaccine-induced immune boosting in shaping long-term immunity and underscore the importance of incorporating immunogenetic context into studies of vaccine response and population-level immunity.

## Data Availability

The raw data supporting the conclusions of this article will be made available by the authors, without undue reservation.
