# Supplementary material for: Longitudinal analysis of hybrid immunity in a Vietnamese COVID-19 cohort demonstrates vaccination-associated broadening of cross-variant neutralization across diverse HLA backgrounds
Source: Front Immunol. 2026 Sep 8;17:1865246. doi: 10.3389/fimmu.2026.1865246 (PMC13593883; doi:10.3389/fimmu.2026.1865246)
Supplement: Supplementary file 1 [file Table1.docx]

**Supplementary Figure 1.** Neutralizing antibody titers stratified by vaccine platform.
Neutralizing antibody (NAb) titers against Wuhan, Alpha, Beta, Gamma, Delta, and Omicron BA.1 variants are shown according to vaccine platform: mRNA vaccine, viral-vector vaccine, and inactivated whole-virus vaccine. Open circles indicate samples collected at 7 months post-infection before vaccination, while open squares indicate samples collected at 15 months post-infection after vaccination. Titers are shown on a log10 scale. Horizontal bars indicate central tendency. Statistical comparisons were performed using the Kruskal–Wallis test with Dunn’s multiple-comparison correction. Asterisks indicate statistically significant differences (*p < 0.05, ***p < 0.001, ****p < 0.0001). Because all participants were vaccine-naïve at the 7-month time point, vaccine-platform grouping at baseline reflects retrospective classification according to the vaccine subsequently received and should not be interpreted as a vaccine effect before vaccination.

**Pre-vaccination**

**(1st collection)**

**Post-vaccination**

**(2nd collection)**

**NAb titer in the first collection (n=189)**

**NAb titer in the second collection (n=68)**

**Asymptomatic**

**Mild/Severe**

**(e)**

**(d)**

**Asymptomatic**

**Mild/Severe**

**(c)**

**(b)**

**(a)**

**Supplementary Figure 2.** Neutralizing antibody (NAb) titers before and after vaccination. PRNT₅₀ titers were measured at 7 months post-infection (n = 189, pre-vaccination) and 15 months post-infection (n = 68, post-vaccination). Panel (c) shows paired samples from the same 63 individuals. Titers were determined against the Wuhan, Alpha, Beta, Gamma, Delta, and Omicron BA1 variants. Panels (d–e) compare NAb titers between asymptomatic and mild/severe groups at both time points. Statistical analysis was performed using one-way ANOVA with Tukey’s multiple-comparison test (p < 0.05). Horizontal lines indicate median values; error bars indicate 95% confidence intervals. Shapes indicate the groups shown in each panel.

**Supplementary Figure 3. Distribution of PRNT50 neutralizing antibody titers against SARS-CoV-2 variants at 7 and 15 months post-infection.**

Stacked bars show the number of participants with PRNT50 titers <20, 20–80, 80–160, and ≥160 against Wuhan, Alpha, Beta, Gamma, Delta, and Omicron BA.1 variants. Panel A shows samples collected at 7 months post-infection before vaccination (n = 189). Panel B shows all available samples collected at the 15-month follow-up, after vaccination in most participants (n = 72). Colors indicate PRNT50 titer categories from low to high. The distribution demonstrates a marked increase in high-titer neutralizing responses at 15 months, while Omicron BA.1 retained a larger proportion of low-titer responses compared with pre-Omicron variants.
