# Supplementary material for: Longitudinal analysis of hybrid immunity in a Vietnamese COVID-19 cohort demonstrates vaccination-associated broadening of cross-variant neutralization across diverse HLA backgrounds
Source: Front Immunol. 2026 Sep 8;17:1865246. doi: 10.3389/fimmu.2026.1865246 (PMC13593883; doi:10.3389/fimmu.2026.1865246)
Supplement: Supplementary file 2 [file Table2.docx]

**Supplementary Table.**

**Supplementary Table 1. Multivariable linear regression analysis of neutralization breadth. Neutralization breadth was defined as the number of variants with PRNT₅₀ ≥20 at 7 months and PRNT₅₀ ≥160 at 15 months. Models were adjusted for vaccination status, age, and sex. Estimates represent regression coefficients with 95% confidence intervals.**

| **Variable** | **7 months Estimate** | **7 months 95% CI** | **7 months p-value** | **15 months Estimate** | **15 months 95% CI** | **15 months p-value** |
| --- | --- | --- | --- | --- | --- | --- |
| Intercept | 1.51 | 0.23 – 2.78 | 0.021 | -0.24 | -0.99 – 0.51 | 0.525 |
| Vaccinated (Yes) | -0.63 | -1.46 – 0.20 | 0.138 | **4.07** | **3.59 – 4.56** | **1.3×10⁻²⁹** |
| Age (per year) | **0.026** | **0.002 – 0.050** | **0.036** | 0.005 | -0.009 – 0.019 | 0.451 |
| Male sex | -0.75 | -1.59 – 0.09 | 0.079 | 0.22 | -0.27 – 0.71 | 0.384 |

**Supplementary Table S2. Distribution of PRNT_50_ neutralizing antibody titers across SARS-CoV-2 variants.**

| **PRNT_50_ titer category** | **Number of participants by PRNT_50_ titer category** | | | | | | | | | | | |
| --- | --- | --- | --- | --- | --- | --- | --- | --- | --- | --- | --- | --- |
|  | **7 months post-infection (n=189)** | | | | | | **15 months post-infection (n=72)** | | | | | |
|  | *Wuhan* | *Alpha* | *Beta* | *Gamma* | *Delta* | *Omicron* | *Wuhan* | *Alpha* | *Beta* | *Gamma* | *Delta* | *Omicron* |
| **<20** | 113 | 87 | 95 | 88 | 159 | 187 | 15 | 7 | 12 | 9 | 12 | 37 |
| **20-80** | 60 | 30 | 74 | 67 | 27 | 2 | 5 | 4 | 11 | 7 | 13 | 12 |
| **80-160** | 13 | 33 | 15 | 20 | 3 | 0 | 5 | 2 | 6 | 16 | 4 | 9 |
| **≥160** | 3 | 39 | 5 | 14 | 0 | 0 | 47 | 59 | 43 | 40 | 43 | 14 |
| **Subtotal ≥20** | **76** | **102** | **94** | **101** | **30** | **2** | **57** | **65** | **60** | **63** | **60** | **35** |
